# Extended-Spectrum β-Lactamase Genes of *Escherichia coli* in Chicken Meat and Humans, the Netherlands

**DOI:** 10.3201/eid1707.110209

**Published:** 2011-07

**Authors:** Ilse Overdevest, Ina Willemsen, Martine Rijnsburger, Andrew Eustace, Li Xu, Peter Hawkey, Max Heck, Paul Savelkoul, Christina Vandenbroucke-Grauls, Kim van der Zwaluw, Xander Huijsdens, Jan Kluytmans

**Affiliations:** Author affiliations: St. Elisabeth Hospital, Tilburg, the Netherlands (I. Overdevest, J. Kluytmans);; Amphia Hospital, Breda, the Netherlands (I. Overdevest, I. Willemsen, J. Kluytmans);; University Medical Centre, Amsterdam, the Netherlands (M. Rijnsburger, P. Savelkoul, C. Vandenbroucke-Grauls, J. Kluytmans);; Heart of England National Health Service Foundation Trust, Birmingham, UK (A. Eustace, L. Xu, P. Hawkey);; University of Birmingham, Birmingham (P. Hawkey);; National Institute of Public Health and Environmental Protection, Bilthoven, the Netherlands (M. Heck, K. van der Zwaluw, X. Hiujsdens)

**Keywords:** Extended-spectrum β-lactamase genes, drug resistance, chicken meat, humans, bacteria, Escherichia coli, Klebsiella, transmission, The Netherlands, research

## Abstract

We determined the prevalence and characteristics of extended-spectrum β-lactamase (ESBL) genes of *Enterobacteriaceae* in retail chicken meat and humans in the Netherlands. Raw meat samples were obtained, and simultaneous cross-sectional surveys of fecal carriage were performed in 4 hospitals in the same area. Human blood cultures from these hospitals that contained ESBL genes were included. A high prevalence of ESBL genes was found in chicken meat (79.8%). Genetic analysis showed that the predominant ESBL genes in chicken meat and human rectal swab specimens were identical. These genes were also frequently found in human blood culture isolates. Typing results of *Escherichia coli* strains showed a high degree of similarity with strains from meat and humans. These findings suggest that the abundant presence of ESBL genes in the food chain may have a profound effect on future treatment options for a wide range of infections caused by gram-negative bacteria.

Infections with drug-resistant bacteria are associated with higher rates of illnesses and deaths, which have a serious effect on costs of health care (*1**,**2*). During the past decade, drug resistance in *Enterobacteriaceae* has increased dramatically worldwide. This increase has been caused mainly by an increased prevalence of extended-spectrum β-lactamase (ESBL)–producing *Enterobacteriaceae* (*3**,**4*) and has increased the use of last-resort antimicrobial drugs (i.e., carbapenems).

ESBL genes are located on plasmids that can be easily transferred between and within bacterial species. Some ESBL genes are mutant derivatives of established plasmid-mediated β-lactamases (e.g., *bla*_TEM/SHV_), and others are mobilized from environmental bacteria (e.g., *bla*_CTX-M_). During the 1990s, most reports on ESBL genes concerned *bla*_TEM/SHV_ types, which were related to cross-infections in hospitals. However, the recent global increase has been caused mainly by *bla*_CTX-M_–type genes. The epidemiology of ESBL genes is changing rapidly and shows marked geographic differences in distribution of genotypes of *bla*_CTX-M_ β-lactamases (*5*). In the United States, the most prevalent drug resistance gene in humans is currently *bla*_CTX-M-15_, which is often associated with a widely distributed variant of *Escherichia coli* O:25b, sequence type 131 (ST131). Bacteria containing ESBL genes are currently a common cause of infections originating in community-dwelling persons without a history of hospitalization, and these organisms can then be introduced into hospitals (*6**–**9*).

Fecal carriage of ESBL genes has been identified as the major reservoir in the environment, but the original source of this colonization has not been clearly identified. Because bacterial species that carry ESBL genes are normal inhabitants of the gastrointestinal tract, food is a potential source of them. The presence of ESBL genes has been clearly documented in food-production animals, especially chickens (*10**,**11*). Drug resistance in animals is caused mainly by the large amount of antimicrobial drugs used in food production. In addition to their presence in farm animals, ESBL genes have been found in retail meat (*12**,**13*). A recent survey of broiler chickens in Great Britain found that *bla*_CTX-M-1_ was the most prevalent ESBL gene (*14*). Although ESBL genes in food-production animals pose a potential threat to humans, Randall et al. concluded that drug resistance genes in chickens (*bla*_CTX-M-1_) differed from the drug resistance genes most frequently found in humans (*bla*_CTX-M-15_) (*14*).

In the Netherlands, use of antimicrobial drugs and associated drug resistance in humans is among the lowest in Europe (*15*). Paradoxally, use of antimicrobial drugs in food-production animals in this country is among the highest in Europe (*16*). Therefore, the Netherlands provides a good setting to monitor spread of drug resistance from an animal reservoir into the human population. This spread was recently exemplified by emergence of livestock-associated methicillin-resistant *Staphylococcus aureus* in pigs and veal calves. This was first reported in the Netherlands in 2004 and has now been reported worldwide (*17*). The aim of our study was to determine the prevalence of ESBL genes in retail meat and hospitalized patients in the Netherlands and to compare ESBL genes and bacterial strains involved.

## Methods

### Meat Survey

During August 17–October 30, 2009, a prospective observational study was conducted in 4 hospitals in the southern part of the Netherlands (Sint Elisabeth Hospital in Tilburg, Twee Steden Hospital in Tilburg, Amphia Hospital in Breda, and Lievensberg Hospital in Bergen op Zoom). Randomly chosen packages of meat from major grocery stores in the region of the 4 participating hospitals were included. Each sample was derived from a different package containing raw and unspiced meat. All samples were incubated for 16–18 h at 37°C in 15 mL of tryptic soy broth (TSB). Subsequently, 100 µL of the initial broth sample was transferred into a second sample of TSB broth containing 8 mg/L vancomycin and 0.25 mg/L cefotaxime. After overnight incubation, 10 µL of the broth was placed on a chromogenic agar plate selective for ESBL (bioMérieux, Marcy l’Etoile, France), and the plates were incubated overnight. Colonies with distinct morphologic appearance were further characterized.

Species and resistance patterns of oxidase-negative, gram-negative rods were determined by using the Vitek2 System (bioMérieux). Phenotypic confirmation of ESBL was performed by using Etest (bioMérieux) for all isolates. A combination of ceftazidime, cefotaxime, and cefepime with and without clavulanic acid was used. If the MIC of >1 of the cephalosporins showed an 8-fold reduction in combination with clavulanic acid, the isolate was considered to be an ESBL producer. If the Etest result was inconclusive, a combination disk diffusion test (Rosco, Taastrup, Denmark) was performed. All tests were performed and interpreted according to the National Guideline for the Laboratory Detection of ESBL (*18*).

### Fecal Carriage Survey

In the 4 hospitals in the same area were the meat had been obtained, 2 consecutive prevalence surveys were performed 3 weeks apart (November 1–December 20, 2009) as part of each hospital’s infection control program. Patients who had positive results for ESBL at the first sampling were excluded from subsequent sampling. These hospitals provide care to ≈1 million persons. Rectal swabs specimens were obtained from all patients admitted to the internal medicine, surgery, urology, pulmonology, and intensive care unit wards. Patients <18 years of age or those who had a colostomy were excluded. Rectal swab specimens were incubated in TSB broth cultures by using broth enrichment as described for the meat samples. Duration of hospitalization on the day of the survey was recorded for all participating patients.

### Blood Cultures

All *E. coli* and *Klebsiella* spp. resistant to cefotaxime, including all strains presumably producing ESBL on the basis of microbiologic results, and isolated from clinical blood cultures, were obtained during July 2008–December 2009 from the 4 study hospitals. Confirmation of ESBL genes was performed as described for meat samples. Blood culture isolates were obtained from individual patients.

### Genetic Characterization of Drug Resistance Genes

Characterization of drug resistance genes in all strains that were phenotypically ESBL producers was conducted 2 ways. First, we tested all isolates for *bla*_CTX-M_ by using denaturing high-performance liquid chromatography as described (*19*). Second, we screened for ESBL genes by using a micro-array (Check-Points, Wageningen, the Netherlands) that was designed to detect single nucleotide polymorphisms in essential *bla*_TEM_ and *bla*_SHV_ genes, variant genes, and *bla*_CTX-M_ group genes (*bla*_CTX-M-1_, *bla*_CTX-M-2_, *bla*_CTX-M-9_, and *bla*_CTX-M-8/25_)_._ The procedure has been reported (*20*). Subsequently, sequencing was performed to further specify ESBL genotypes. On the basis of micro-array results, *bla*_SHV_, *bla*_TEM_, and *bla*_CTX-M_ genes were amplified by PCR and specific primers. PCR amplicons were selected and sequenced after purification (Agencourt Ampure; Beckman Coulter, Leiden, the Netherlands). Sequence analysis and alignments were performed by using Bionumerics 6.01 software (Applied Maths, Sint-Martens-Latem, Belgium), the BLAST program (http://blast.ncbi.nlm.nih.gov/Blast.cgi), and information from the Lahey Clinic (Burlington, MA, USA) (www.lahey.org/studies). If results of denaturing high-perfomance liquid chromatography and DNA sequence were discordant, the DNA sequence was used as the correct result.

### Multilocus Sequence Typing of *E*. *coli* Strains

All *E. coli* isolates from meat, rectal swab specimens, and blood cultures were typed by using multilocus sequence typing (MLST) as described by Wirth et al. (*21*). If patient or meat samples contained >1 morphologically distinct ESBL-producing *E. coli* strain, all strains were included in the MLST analysis.

### Statistical Analysis

Data were analyzed by using SPSS version 18 software (SPSS, IBM, Somers, NY, USA). Univariate analysis was performed for calculation of difference in prevalences by using the χ^2^ test. One sample could contain >1 strain because morphologically distinct colonies with different drug resistance genes or a different MLST result were all included in the final analysis.

## Results

### Meat Survey

A total of 262 fresh meat samples were included in this study (mean weight 11.9 g). The type of meat was chicken (n = 89, 34.0%), beef (n = 85, 32.4%), pork (n = 57, 21.8%), mixed or ground meat (n = 22, 8.4%), and other types of meat (n = 9, 3.4%). Phenotypic screening initially identified 112 samples containing possible ESBL producers. Genotypic confirmation identified 79 (30.2%) ESBL-producing samples. Some samples contained >1 type of ESBL gene. Prevalence of ESBL genes differed among the 4 meat groups: 71 (79.8%) in chicken, 4 (4.7%) in beef, 1 (1.8%) in pork, 2 (9.1%) in mixed or ground meat, and 1 (11.1%) in other types of meat. ESBL gene prevalence was significantly higher in chicken (p<0.001 for all comparisons with other meat types).

### Fecal Carriage Survey

A total of 927 rectal swab specimens were obtained from 876 patients (461 male patients and 415 female patients, mean ± SD age 65.7 ± 16.8 years). Phenotypic screening identified 59 patients as possibly being infected with ESBL-producing *E*. *coli*. Confirmatory test results for ESBL genes were inconclusive for 2 samples and excluded (no inconclusive results were obtained with meat or blood cultures). A total of 45 (4.9%) samples contained confirmed ESBL genes.

### Blood Cultures

Thirty-one clinical blood cultures suspected of containing ESBL genes on the basis of phenotypic screening were available for further analysis. Genetic characterization confirmed that 23 (74.2%) samples contained ESBL genes.

### ESBL-producing Bacterial Species

Sixty-eight (76.8%) chicken meat samples contained ESBL-producing *E. coli*, 6 (7.7%) contained ESBL-producing *Klebsiella* spp., and 4 (5.1%) contained other ESBL-producing species. Of the 8 types found in other meat, all were ESBL-producing *E. coli.* Rectal swab specimens of hospitalized patients showed that 39 (69.6%) patients had *E. coli*, 11 (19.6%) had *Klebsiella* spp., and 8 (10.7%) had other bacterial species. Blood cultures showed that 16 (64.0%) patients had *E. coli* and 9 (36.0%) had *Klebsiella* spp.

### Drug Resistance Genes

**Figure 1:**
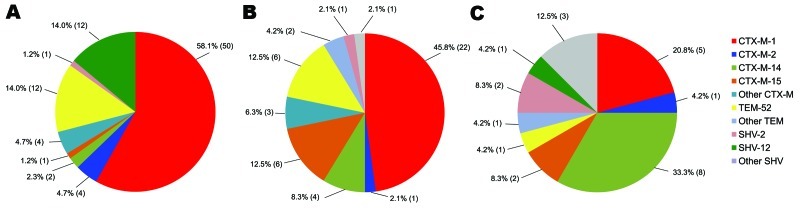
Distribution of extended-spectrum β-lactamase genes in chicken meat (A), human rectal swabs (B), and human blood cultures (C), the Netherlands. Values in parentheses are no. positive.

The distribution of drug resistance genes from various sources is shown in Figure 1. The predominant ESBL genotype in chicken meat was *bla*_CTX-M-1_ (n = 50, 58.1%). This genotype was also the most frequent ESBL genotype in rectal swab specimens (n = 22, 45.8%) and the second most common in blood cultures (n = 5, 20.8%). In chicken meat, *bla*_CTX-M-1_ (n = 50, 58.1%) was the most common genotype, followed by *bla*_TEM-52_ (n = 12, 14.0%) and *bla*_SHV-12_ (n = 12, 14.0%). In other types of meat, 5 (62.5%) of 8 ESBL genotypes were *bla*_CTX-M-1_. In rectal swab specimens of hospitalized patients, *bla*_CTX-M-1_ (n = 22, 45.8%) was the most common genotype, followed by *bla*_CTX-M-15_ (n = 8, 16.7%) and *bla*_TEM-52_ (n = 6, 12.5%). In blood cultures, *bla*_CTX-M-14_ was the predominant genotype (n = 8, 33.3%).

### Epidemiology of Patients and Drug Resistance Genes

When we compared the most prevalent drug resistance genes in 346 patients who had been hospitalized <48 h at the time of screening with 581 patients who had been hospitalized >48 h, prevalence was similar for *bla*_CTX-M-1_ (2.3% and 2.4%, respectively) and for TEM-52 (0.6% and 0.7%, respectively) in the 2 groups. Prevalence of *bla*_CTX-M-15_ was 4× higher in the group that was hospitalized longer (0.3% and 1.2%, respectively; p = 0.27). There was only 1 possible cluster of cases (same resistance gene at the same time at the same ward), which involved 3 patients with *bla*_CTX-M-15_. One patient was infected with *E. coli* and 2 were infected with *K. pneumoniae*.

### MLST of *E*. *coli*

**Figure 2:**
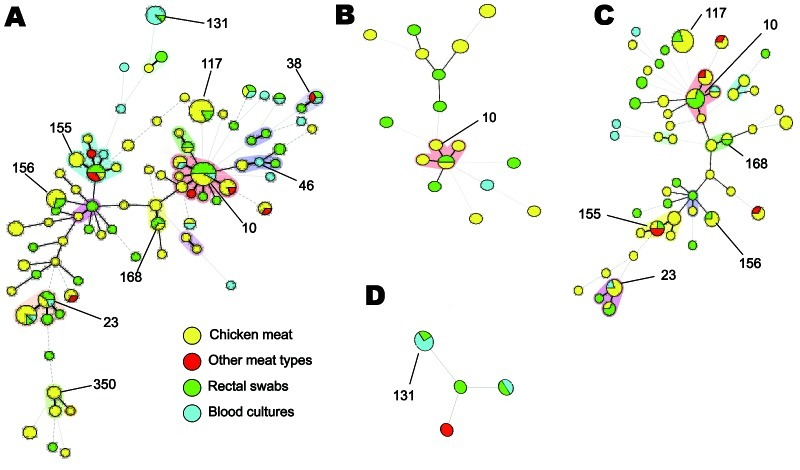
Multilocus sequence typing patterns of *Escherichia coli* from chicken meat, other meat types, human rectal swabs, and human blood cultures, the Netherlands. A) All *E*. *coli* containing extended-spectrum β-lactamase genes; B) *E*. *coli* containing *bla*_TEM-52_; C) *E. coli* containing *bla*_CTX-M-1_; D) *E. coli* containing *bla*_CTX-M-15_. Major sequence types are shown as numbers. Black connecting lines indicate single-locus variants; gray connecting lines indicate double-locus variants; dashed connecting lines indicate strains with >3 loci that are different; and shadowing indicates that >2 sequence types belong to 1 complex.

MLST results of 158 ESBL-positive *E. coli* strains isolated from chicken meat, other meat types, rectal swab specimens, and blood cultures are shown in Figure 2. *E. coli* containing ESBL genes showed a heterogeneous population that contained several clusters. Most clusters contained strains isolated from meat and humans. All but 1 of the ESBL-producing strains from other meat types clustered with strains from chicken meat. Twenty-five (56.8%) of 44 strains from rectal swab specimens and 9 (56.3%) of 16 strains from blood cultures clustered with strains from chicken meat.

MLST results for strains with *bla*_CTX-M-1_, *bla*_CTX-M-15_, and *bla*_TEM-52_ are shown in Figure 2. Genotypes *bla*_CTX-M-1_ and *bla*_TEM-52_ showed a heterogeneous population and clusters containing strains from humans and meat. *E. coli* harboring *bla*_CTX-M-15_ was found less frequently, and no clusters with human-derived and meat-derived strains were observed. The widely disseminated ST131 clone was found in human samples only: 4 times in combination with *bla*_CTX-M-15_, twice with *bla*_CTX-M-14_, and once with *bla*_SHV-12_.

## Discussion

ESBL genes were found in a high (79.8%) proportion of retail chicken meat samples in the Netherlands. A comparison of ESBL-producing *Enterobacteriaceae* derived from meat and hospitalized patients showed a high degree of similarity of resistance genes and MLST patterns. Genotype *bla*_CTX-M-1_ was the most frequent drug resistance gene in chicken meat and humans and the second most frequent in blood cultures. Other meat types contained similar drug resistance genes, but the prevalence of ESBL genes was much lower. It is unclear whether ESBL genes in other meat types are related to a reservoir in food-production animals or contamination at meat-processing facilities. An extensive reservoir of ESBL genes on farms was repeatedly shown in poultry (*10**,**11*). Our findings suggest a relationship between contamination of chicken meat with drug-resistant bacteria and appearance of ESBL genes in humans in the Netherlands. This relationship was further supported by genomic comparison of strains detected in chicken meat with those detected in human fecal specimens. MLST showed that most *E. coli* strains harboring *bla*_CTX-M-1_ or *bla*_TEM-52_ from humans and meat belong to clusters containing strains from both sources. These findings suggest a relationship between contamination of chicken meat and appearance of ESBL genes in humans in the Netherlands.

The high prevalence of ESBL genes in chicken meat is consistent with findings of other investigators. Doi et al. reported that 67% of retail meat samples in Seville, Spain, contained ESBL or ESBL-like resistance genes (*12*). A survey of imported raw chicken in the United Kingdom reported ESBL genes in 10 of 27 samples (*13*). The authors concluded that ESBL genes in meat pose a potential threat to humans, but that the most prevalent ESBL genotype in humans in the United Kingdom (*bla*_CTX-M-15_) was not found in imported meat.

In our study, we found a high degree of similarity between drug resistance genes in humans and retail meat. A possible explanation for this finding is that in the Netherlands, where drug resistance in bacterial isolates in humans is less frequent (*15*) and cross-transmission in hospitals is controlled effectively (*22*), the role of acquiring drug-resistant strains from food is more easily detected. In addition, the Netherlands is one of the highest users of antimicrobial agents in food-production animals (*16*), which results in high rates of drug resistance among these animals. A report by the Veterinary Antibiotic Usage and Resistance Surveillance Working Group showed that cefotaxime-resistant *E. coli* in poultry meat in the Netherlands has emerged since 2005 (*23*). This finding coincides with the increase in third-generation cephalosporin-resistance in *E. coli* and *K. pneumonia* bacteria in invasive infections in humans reported by the European Antimicrobial Resistance Surveillance Network (*24*).

We performed a prevalence survey among hospitalized patients to determine the size and nature of the reservoir of ESBL genes. Nearly 5% of all hospitalized patients were carriers of ESBL genes. It is difficult to put this rate into context because no screening studies have been conducted in the Netherlands. A large study in Chicago, Illinois, USA, found that during 2000–2005, the rate of ESBL gene carriage among high-risk, hospitalized patients increased from 1.3% to 3.2%, and bacteremia developed in 8.5% of all previously identified ESBL gene carriers during hospitalization (*25*). In Spain, an increase was also observed in fecal carriage in hospitalized patients from 0.3% in 1991 to 11.8% in 2003 (*7*).

The rate we observed in 2009 in the Netherlands was lower than rates in Spain in 2003, which would be expected because of low antimicrobial drug resistance rates in the Netherlands (*24*). Conversely, a prevalence of 5% extrapolated to the Dutch population in 2011 (16,700,000 inhabitants) would indicate that currently >800,000 persons in the Netherlands are colonized with ESBL-producing bacteria. Because use of antimicrobial drugs in the health care setting in the Netherlands is low and has not changed during the past decade, alternative factors for increased drug resistance are not known. An indication for the role of a community reservoir is the prevalence of drug resistance genes in patients who had been hospitalized <48 h and those hospitalized >48 h. Drug resistance genes that are associated with a proposed food reservoir (*26*) (*bla*_CTX-M-1_ and *bla*_TEM-52_) were already present at hospitalization. However, *bla*_CTX-M-15_, which is reported to be associated with health care settings (*26*), had a higher, albeit not significant, prevalence in the group who had been in the hospital >48 h.

Considering what is known about the epidemiology of *E. coli*, the abundance of ESBL genes in chicken meat is a likely explanation for current findings in humans. Although there are extensive campaigns promoting safe handling of chicken meat during processing, enteric pathogens are frequently transferred to humans and pose a continuous public health threat (*27*). Johnson et al. studied geographically and temporally matched *E. coli* isolates from humans and poultry (*28*). Drug-susceptible *E. coli* isolates from humans differed from drug-resistant isolates from humans and from isolates in poultry irrespective of their drug resistance pattern. Drug-susceptible isolates from poultry were similar to drug-resistant isolates of poultry and humans. Their conclusion was that drug-resistant human fecal *E. coli* isolates likely originate from poultry, whereas drug-resistant *E. coli* isolates from poultry likely originate from susceptible precursors in poultry. In vitro experimental support for our hypothesis comes from a recent study showing transfer of a *bla*_TEM-52_–carrying plasmid from an avian *E. coli* strain to 2 human *E. coli* strains in a continuous flow culture model (*29*).

Emergence of ESBL genes in poultry has been associated with use of third-generation cephalosporins (particularly ceftiofur) in chickens. In Canada, a strong correlation was found between incidence of ceftiofur-resistant *Samonella enterica* serovar Heidelberg in humans and retail chickens (*30*). Use of ceftiofur in animals was stopped voluntarily, and ceftiofur resistance rates subsequently decreased in retail chicken meat and humans. After partial reintroduction of this drug, resistance rates in poultry and humans increased, providing further evidence for a zoonotic source of ESBL genes.

Overrepresentation of ST131 in blood cultures confirms the virulent properties attributed to this clone. Typically, ST131 is found in association with *bla*_CTX-M-15_. In our survey, ST131 strains were associated with 3 drug resistance genes. This finding indicates that this virulent clone also acquires other drug resistance genes. We did not find ST131 in the chicken meat samples, which is reassuring at this time. However, other studies have recently identified ST131 in poultry and retail meat (*31**,**32*). These findings confirm that virulent clones of *E. coli* are capable of crossing species barriers between humans and animals. In addition, mobile drug resistance genes also cross this barrier and are likely to accelerate dissemination of drug resistance between animals and humans.

We conclude that the high rate of ESBL contamination of retail chicken meat in the Netherlands, which involves many of the same ESBL genes present in colonized and infected humans, is a plausible source of the recent increase of ESBL genes in the Netherlands. The similarity of *E. coli* strains and predominant drug resistance genes in meat and humans provides circumstantial evidence for an animal reservoir for a substantial part of ESBL genes found in humans. The threat of the high rate of antimicrobial drug use in food-production animals and associated emergence of drug resistance in zoonotic pathogens has been recognized for decades. Our group and others found that most samples of retail chicken meat contain transmissible drug resistance genes in bacterial species that are part of the normal human intestinal flora. This finding may have a profound effect on future treatment options for a wide range of infections with gram-negative bacteria.
